# Bis[2-(morpholinometh­yl)phen­yl]phenyl­phosphane

**DOI:** 10.1107/S1600536809048946

**Published:** 2009-11-21

**Authors:** Ancuta Covaci, Richard A. Varga, Cristian Silvestru

**Affiliations:** a Faculty of Chemistry and Chemical Engineering, Babes-Bolyai University, Arany Janos Str. no. 11, RO-400028, Cluj Napoca, Romania

## Abstract

The title compound, C_28_H_33_N_2_O_2_P, contains a penta­coordinated P atom as a result of the weak N→P intra­molecular inter­actions, with three C atoms, two N atoms and the lone pair arranged in a dicapped pseudo-tetra­hedral geometry. The morpholine rings exhibit an almost ideal chair conformation. In the crystal, two weak C—H⋯O hydrogen-bond inter­actions link the mol­ecules in layers stacked along the *a* axis; there are no further inter­actions between the layers.

## Related literature

For related structures, see Chuit *et al.* (1993 ▶); Copolovici, *et al.* (2007 ▶); Copolovici, Silvestru, Isaia *et al.* (2008 ▶); Copolovici, Silvestru & Varga (2008 ▶). For the use of phosphines containing organic groups with pendant arms as ligands in the coordination chemistry, see Alonso *et al.* (2003 ▶), Brammer *et al.* (2000 ▶), de Graaf *et al.* (1988 ▶), Kapteijn *et al.* (1996 ▶), Fierro-Arias *et al.* (2005 ▶), Pfeiffer *et al.* (2000 ▶)]. For van der Waals radii, see: Emsley (1994 ▶).
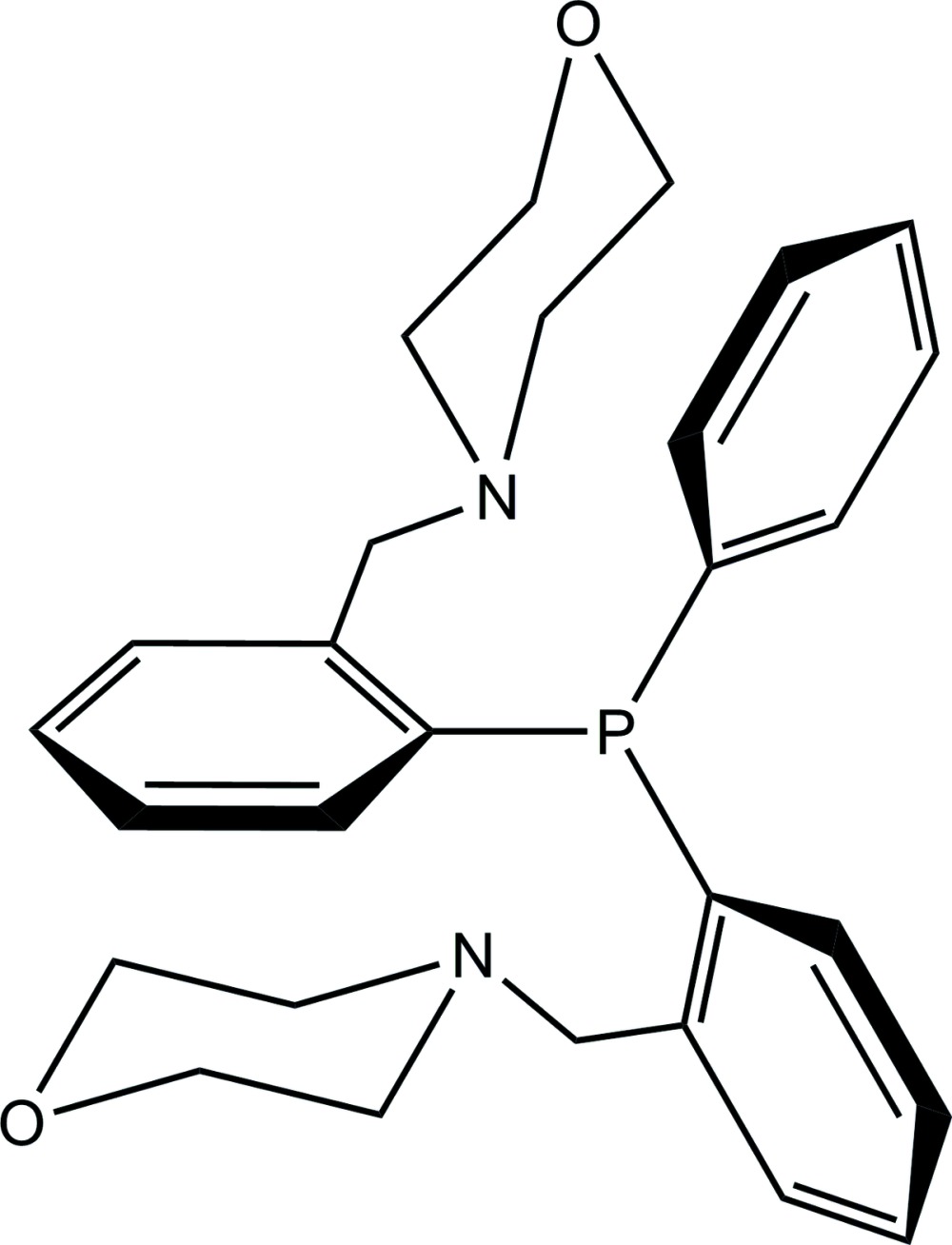


## Experimental

### 

#### Crystal data

C_28_H_33_N_2_O_2_P
*M* *_r_* = 460.53
Monoclinic, 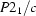

*a* = 14.640 (7) Å
*b* = 11.656 (5) Å
*c* = 14.998 (7) Å
β = 101.950 (9)°
*V* = 2504 (2) Å^3^
*Z* = 4
Mo *K*α radiation
μ = 0.14 mm^−1^
*T* = 297 K
0.30 × 0.26 × 0.12 mm

#### Data collection

Bruker SMART APEX CCD area-detector diffractometer
Absorption correction: multi-scan (*SADABS*; Bruker, 2000 ▶) *T*_min_ = 0.960, *T*_max_ = 0.984
17694 measured reflections
4412 independent reflections
3184 reflections with *I* > 2σ(*I*)
*R*_int_ = 0.099

#### Refinement

*R*[*F* ^2^ > 2σ(*F* ^2^)] = 0.112
*wR*(*F* ^2^) = 0.207
*S* = 1.23
4412 reflections
298 parameters
H-atom parameters constrained
Δρ_max_ = 0.47 e Å^−3^
Δρ_min_ = −0.27 e Å^−3^

### 

Data collection: *SMART* (Bruker, 2000 ▶); cell refinement: *SAINT-Plus* (Bruker, 2001 ▶); data reduction: *SAINT-Plus*; program(s) used to solve structure: *SHELXS97* (Sheldrick, 2008 ▶); program(s) used to refine structure: *SHELXL97* (Sheldrick, 2008 ▶); molecular graphics: *DIAMOND* 3 (Brandenburg & Putz, 2006 ▶) and *ORTEP-3* (Farrugia, 1997 ▶); software used to prepare material for publication: *publCIF* (Westrip, 2009 ▶).

## Supplementary Material

Crystal structure: contains datablocks I, global. DOI: 10.1107/S1600536809048946/bq2178sup1.cif

Structure factors: contains datablocks I. DOI: 10.1107/S1600536809048946/bq2178Isup2.hkl

Additional supplementary materials:  crystallographic information; 3D view; checkCIF report

## Figures and Tables

**Table 1:** Hydrogen-bond geometry (Å, °)

*D*—H⋯*A*	*D*—H	H⋯*A*	*D*⋯*A*	*D*—H⋯*A*
C25—H25⋯O1^i^	0.93	2.46	3.370 (8)	166
C11—H11*B*⋯O2^ii^	0.97	2.55	3.426 (6)	151

Symmetry codes: (i) 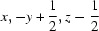; (ii) 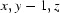.

**Fig. 1.**
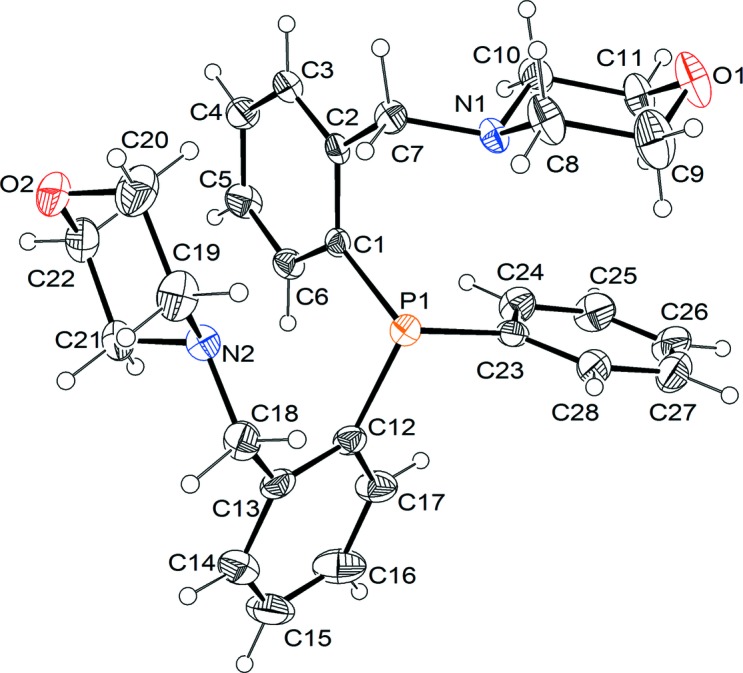
: The molecular structure of the title compound showing 30% probability displacement ellipsoids and the atom-numbering scheme.

**Fig. 2.**
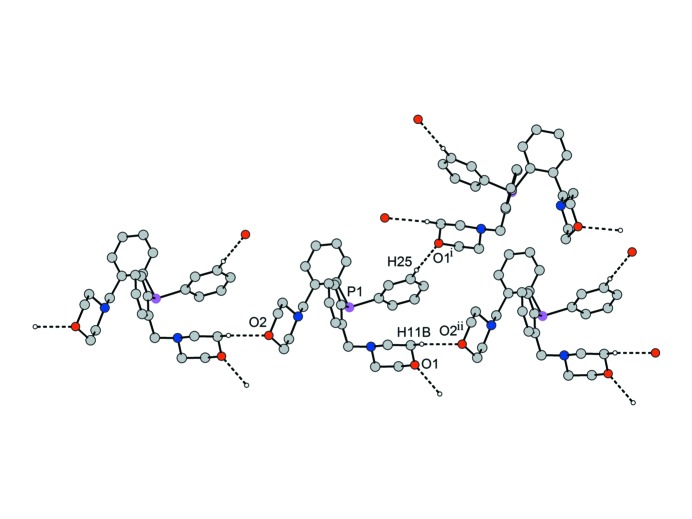
: Hydrogen bonds in the title compound (dashed lines; only H atoms involved in interactions are shown). Symmetry codes: (i) x, -y+1/2, z-1/2; (ii) x, y-1, z.

**Fig. 3.**
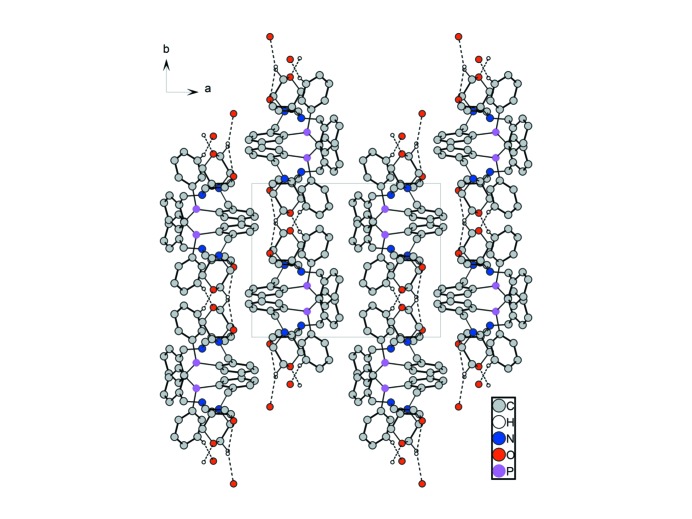
: Molecular packing as viewed along the c axis. Hydrogen bonds are shown as dashed lines; only H atoms involved in interactions are shown.

## supplementary crystallographic information

### Comment

Phosphines containing organic groups with pendant arms, *e.g. *PPh_n_[C_6_H_4_(CH_2_NMe_2_)-2]_3-n_, were successfully used as ligands in the coordination chemistry of various transition metals [Co (Brammer *et al.*, 2000), Rh (Alonso *et al.*, 2003), Pd (de Graaf *et al.*, 1988; Kapteijn *et al.*, 1996; Fierro-Arias *et al.*, 2005), Pt (Pfeiffer *et al.*, 2000)]. In order to extend this class of potential phosphine ligands we decided to investigate other related compounds and here we report on the molecular structure of PPh[C_6_H_4_{CH_2_N(CH_2_CH_2_)_2_O}-2]_2_.

The structure of (I) with its atomic numbering scheme is depicted in Figure 1. The N atoms from the two morphilinyl pendant arms form weak intramolecular interactions with the central phosphorus atom [N1···P1 = 3.038 (4) and N2···P1 = 3.105 (4) Å; c.f. sums of the covalent radii, Σ*r*_cov_(*P*,*N*) 1.80 Å, and van der Waals radii, Σ*r*_vdW_(*P*,*N*) 3.44 Å (Emsley, 1994)]. The magnitude of the N→P interactions is similar to the ones present in tris[2-(dimethylaminomethyl)phenyl]phosphane (Chuit *et al.*, 1993). Taking into account these intramolecular interactions a dicapped *pseudo*-tetrahedron can be considered around the phosphorus, with the three carbon atoms and the phosphorus lone pair describing the tetrahedral skeleton.

An almost ideal *chair *conformation was observed for both morpholinyl groups with torsion angles [C8—N1—C11—C10 = 56.5 (6)°, C10—O1—C9—C8 = -57.9 (6)°, C19—N2—C21—C22 = 56.7 (6)° and C22—O2—C20—C19 = -58.1 (6)°] similar with those found in 4-benzylmorpholin-4-ium chloride (Copolovici *et al.*, 2007), tris[2-(morpholin-4-ylmethyl)phenyl-*κ*^2^*C*^1^,*N*]antimony(III) (Copolovici, Silvestru & Varga (2008) and in di-*µ*-chlorido-bis{[2-(morpholin-4-ylmethyl)phenyl-*k*^2^*C*^1^,*N*]palladium(II)} (Copolovici, Silvestru, Isaia *et al.* (2008).

Weak hydrogen bonds between one morpholinyl oxygen atom and an aromatic C—H [H25···O1^i^ = 2.46 Å; symmetry code: (i) *x*, -*y* + 1/2, *z* - 1/2] and between the other morpholinyl oxygen and a CH_2_ hydrogen [H11B···O2^ii^ = 2.55 Å; symmetry code: (ii) *x*, *y* - 1, *z*] (Figure 2) give rise to a bidimensional layer along the *bc* plane. The layers are stacked along the *a* axis, with no further interactions (Figure 3).

### Experimental

To a solution of the [2-{O(CH_2_CH_2_)_2_NCH_2_}C_6_H_4_]Li (2.72 g, 14 mmol) in cold thf (-70 °C) was added dropwise a solution of PPhCl_2_(1.01 ml, ρ = 1.319 g/ml, 7 mmol) in thf. The reaction mixture was stirred at -70 °C for additional 2 h, then it was allowed to reach the room temperature and the solvent was removed under vacuum. The obtained oily product was extracted with CH_2_Cl_2_. The solid residue was filtered off and the solvent was removed in vacuum. The remaining viscous oil solidified on addition of hexane. The title compound was isolated as a white solid. Colorless crystals suitable for X-ray diffraction studies were obtained by slow diffusion of hexane into a CH_2_Cl_2_ solution of the title compound (1:1 *v/v* ratio) (yield: 2.76 g, 81%; m.p. 89 °C).

### Refinement

All H atoms were placed in calculated positions (C—H = 0.93–0.97 Å) and treated using a riding model with *U*_iso_= 1.2*U*_eq_(C). The R factor is 0.112 due to the crystal quality and because the measurement was made at room temperature. We tried several times to grow quality crystals and measured 4 different ones but only the one submitted was acceptable.

### Crystal data

**Table:**

C_28_H_33_N_2_O_2_P	*F*(000) = 984
*M**_r_* = 460.53	*D*_x_ = 1.222 Mg m^−^^3^
Monoclinic, *P*2_1_/*c*	Mo *K*α radiation, λ = 0.71073 Å
Hall symbol: -P 2ybc	Cell parameters from 2411 reflections
*a* = 14.640 (7) Å	θ = 2.2–20.0°
*b* = 11.656 (5) Å	µ = 0.14 mm^−^^1^
*c* = 14.998 (7) Å	*T* = 297 K
β = 101.950 (9)°	Block, colourless
*V* = 2504 (2) Å^3^	0.30 × 0.26 × 0.12 mm
*Z* = 4	

### Data collection

**Table:**

Bruker SMART APEX CCD area-detector diffractometer	4412 independent reflections
Radiation source: fine-focus sealed tube	3184 reflections with *I* > 2σ(*I*)
graphite	*R*_int_ = 0.099
phi and ω scans	θ_max_ = 25.0°, θ_min_ = 2.2°
Absorption correction: multi-scan (*SADABS*; Bruker, 2000)	*h* = −17→17
*T*_min_ = 0.960, *T*_max_ = 0.984	*k* = −13→13
17694 measured reflections	*l* = −17→17

### Refinement

**Table:**

Refinement on *F*^2^	Primary atom site location: structure-invariant direct methods
Least-squares matrix: full	Secondary atom site location: difference Fourier map
*R*[*F*^2^ > 2σ(*F*^2^)] = 0.112	Hydrogen site location: inferred from neighbouring sites
*wR*(*F*^2^) = 0.207	H-atom parameters constrained
*S* = 1.23	*w* = 1/[σ^2^(*F*_o_^2^) + (0.0459*P*)^2^ + 3.678*P*] where *P* = (*F*_o_^2^ + 2*F*_c_^2^)/3
4412 reflections	(Δ/σ)_max_ < 0.001
298 parameters	Δρ_max_ = 0.47 e Å^−^^3^
0 restraints	Δρ_min_ = −0.27 e Å^−^^3^

### Special details

**Table:**

Geometry. All e.s.d.'s (except the e.s.d. in the dihedral angle between two l.s. planes) are estimated using the full covariance matrix. The cell e.s.d.'s are taken into account individually in the estimation of e.s.d.'s in distances, angles and torsion angles; correlations between e.s.d.'s in cell parameters are only used when they are defined by crystal symmetry. An approximate (isotropic) treatment of cell e.s.d.'s is used for estimating e.s.d.'s involving l.s. planes.
Refinement. Refinement of *F*^2^ against ALL reflections. The weighted *R*-factor *wR* and goodness of fit *S* are based on *F*^2^, conventional *R*-factors *R* are based on *F*, with *F* set to zero for negative *F*^2^. The threshold expression of *F*^2^ > σ(*F*^2^) is used only for calculating *R*-factors(gt) *etc*. and is not relevant to the choice of reflections for refinement. *R*-factors based on *F*^2^ are statistically about twice as large as those based on *F*, and *R*- factors based on ALL data will be even larger.

### Fractional atomic coordinates and isotropic or equivalent isotropic displacement parameters (Å^2^)

**Table:**

	*x*	*y*	*z*	*U*_iso_*/*U*_eq_	
C1	0.8291 (3)	0.6869 (4)	0.0252 (3)	0.0286 (11)	
C2	0.8999 (3)	0.6704 (4)	0.1032 (3)	0.0331 (11)	
C3	0.9912 (4)	0.6862 (4)	0.0965 (4)	0.0433 (14)	
H3	1.0382	0.6763	0.1481	0.052*	
C4	1.0150 (4)	0.7158 (5)	0.0161 (4)	0.0469 (14)	
H4	1.0774	0.7250	0.0132	0.056*	
C5	0.9466 (4)	0.7320 (5)	−0.0602 (4)	0.0475 (15)	
H5	0.9623	0.7521	−0.1152	0.057*	
C6	0.8551 (3)	0.7185 (4)	−0.0550 (3)	0.0360 (12)	
H6	0.8090	0.7308	−0.1069	0.043*	
C7	0.8755 (3)	0.6394 (4)	0.1929 (3)	0.0358 (12)	
H7A	0.9324	0.6350	0.2392	0.043*	
H7B	0.8370	0.6997	0.2103	0.043*	
C8	0.7893 (4)	0.5140 (5)	0.2711 (4)	0.0552 (16)	
H8A	0.7469	0.5761	0.2772	0.066*	
H8B	0.8401	0.5149	0.3242	0.066*	
C9	0.7389 (5)	0.4023 (5)	0.2665 (5)	0.070 (2)	
H9A	0.7151	0.3923	0.3218	0.084*	
H9B	0.6859	0.4038	0.2155	0.084*	
C10	0.8860 (4)	0.4335 (4)	0.1789 (4)	0.0460 (14)	
H10A	0.9397	0.4319	0.2291	0.055*	
H10B	0.9084	0.4420	0.1227	0.055*	
C11	0.8324 (4)	0.3235 (4)	0.1766 (4)	0.0540 (16)	
H11A	0.7811	0.3238	0.1240	0.065*	
H11B	0.8730	0.2597	0.1699	0.065*	
C12	0.6424 (3)	0.7480 (4)	−0.0629 (3)	0.0355 (12)	
C13	0.6155 (3)	0.8617 (4)	−0.0499 (4)	0.0397 (13)	
C14	0.5645 (4)	0.9221 (5)	−0.1217 (4)	0.0568 (16)	
H14	0.5491	0.9979	−0.1125	0.068*	
C15	0.5352 (4)	0.8745 (6)	−0.2073 (5)	0.070 (2)	
H15	0.4995	0.9165	−0.2547	0.085*	
C16	0.5607 (4)	0.7625 (7)	−0.2202 (4)	0.072 (2)	
H16	0.5423	0.7280	−0.2770	0.086*	
C17	0.6126 (4)	0.7027 (5)	−0.1497 (4)	0.0507 (15)	
H17	0.6291	0.6276	−0.1602	0.061*	
C18	0.6392 (4)	0.9177 (5)	0.0427 (4)	0.0457 (14)	
H18A	0.6131	0.9945	0.0379	0.055*	
H18B	0.6094	0.8748	0.0842	0.055*	
C19	0.7540 (4)	0.9681 (5)	0.1751 (4)	0.0569 (16)	
H19A	0.7243	0.9175	0.2118	0.068*	
H19B	0.7264	1.0437	0.1757	0.068*	
C20	0.8568 (4)	0.9748 (6)	0.2152 (4)	0.0660 (18)	
H20A	0.8661	1.0056	0.2765	0.079*	
H20B	0.8833	0.8983	0.2190	0.079*	
C21	0.7873 (4)	0.9979 (5)	0.0286 (4)	0.0478 (14)	
H21A	0.7616	1.0749	0.0252	0.057*	
H21B	0.7791	0.9682	−0.0330	0.057*	
C22	0.8891 (4)	1.0021 (5)	0.0712 (4)	0.0543 (16)	
H22A	0.9154	0.9256	0.0720	0.065*	
H22B	0.9210	1.0509	0.0350	0.065*	
C23	0.6850 (3)	0.5187 (4)	−0.0066 (3)	0.0347 (12)	
C24	0.7352 (4)	0.4612 (5)	−0.0615 (4)	0.0481 (14)	
H24	0.7852	0.4978	−0.0788	0.058*	
C25	0.7123 (4)	0.3509 (5)	−0.0911 (4)	0.0590 (17)	
H25	0.7456	0.3143	−0.1293	0.071*	
C26	0.6405 (4)	0.2956 (5)	−0.0640 (5)	0.0633 (19)	
H26	0.6251	0.2210	−0.0835	0.076*	
C27	0.5907 (4)	0.3504 (5)	−0.0075 (5)	0.0669 (19)	
H27	0.5421	0.3125	0.0114	0.080*	
C28	0.6129 (4)	0.4608 (5)	0.0209 (4)	0.0481 (14)	
H28	0.5792	0.4971	0.0589	0.058*	
N1	0.8259 (3)	0.5307 (3)	0.1898 (3)	0.0336 (10)	
N2	0.7376 (3)	0.9253 (3)	0.0814 (3)	0.0354 (10)	
O1	0.7968 (3)	0.3077 (3)	0.2563 (3)	0.0681 (13)	
O2	0.9036 (3)	1.0454 (3)	0.1617 (3)	0.0595 (11)	
P1	0.70675 (9)	0.66657 (11)	0.03556 (9)	0.0311 (3)	

### Atomic displacement parameters (Å^2^)

**Table:**

	*U*^11^	*U*^22^	*U*^33^	*U*^12^	*U*^13^	*U*^23^
C1	0.028 (2)	0.021 (3)	0.037 (3)	0.001 (2)	0.008 (2)	0.000 (2)
C2	0.040 (3)	0.020 (3)	0.037 (3)	−0.002 (2)	0.002 (2)	0.005 (2)
C3	0.045 (3)	0.030 (3)	0.051 (4)	0.001 (2)	0.000 (3)	0.007 (3)
C4	0.028 (3)	0.050 (4)	0.064 (4)	0.002 (3)	0.014 (3)	0.014 (3)
C5	0.044 (3)	0.060 (4)	0.044 (3)	0.005 (3)	0.021 (3)	0.018 (3)
C6	0.039 (3)	0.038 (3)	0.032 (3)	0.002 (2)	0.007 (2)	0.005 (2)
C7	0.048 (3)	0.031 (3)	0.025 (3)	−0.003 (2)	0.001 (2)	−0.007 (2)
C8	0.083 (4)	0.040 (3)	0.051 (4)	0.007 (3)	0.032 (3)	0.009 (3)
C9	0.099 (5)	0.059 (4)	0.068 (5)	0.001 (4)	0.054 (4)	0.010 (4)
C10	0.055 (4)	0.036 (3)	0.046 (3)	0.002 (3)	0.007 (3)	0.002 (3)
C11	0.076 (4)	0.027 (3)	0.062 (4)	0.001 (3)	0.022 (3)	0.004 (3)
C12	0.026 (3)	0.037 (3)	0.043 (3)	0.003 (2)	0.007 (2)	−0.004 (3)
C13	0.023 (3)	0.045 (3)	0.050 (3)	−0.001 (2)	0.007 (2)	−0.003 (3)
C14	0.049 (4)	0.050 (4)	0.068 (4)	0.013 (3)	0.004 (3)	0.004 (3)
C15	0.050 (4)	0.082 (5)	0.071 (5)	0.018 (4)	−0.005 (3)	0.020 (4)
C16	0.060 (4)	0.100 (6)	0.047 (4)	0.015 (4)	−0.008 (3)	−0.006 (4)
C17	0.047 (3)	0.055 (4)	0.044 (4)	0.008 (3)	−0.005 (3)	−0.011 (3)
C18	0.042 (3)	0.037 (3)	0.062 (4)	0.006 (3)	0.019 (3)	−0.007 (3)
C19	0.066 (4)	0.058 (4)	0.053 (4)	−0.010 (3)	0.025 (3)	−0.007 (3)
C20	0.077 (5)	0.067 (4)	0.053 (4)	−0.022 (4)	0.012 (4)	−0.012 (3)
C21	0.052 (4)	0.037 (3)	0.056 (4)	−0.005 (3)	0.013 (3)	0.007 (3)
C22	0.057 (4)	0.040 (4)	0.068 (4)	−0.007 (3)	0.020 (3)	−0.002 (3)
C23	0.031 (3)	0.035 (3)	0.035 (3)	0.005 (2)	−0.001 (2)	0.002 (2)
C24	0.049 (3)	0.047 (4)	0.049 (4)	−0.002 (3)	0.010 (3)	−0.011 (3)
C25	0.056 (4)	0.051 (4)	0.068 (4)	0.005 (3)	0.009 (3)	−0.023 (3)
C26	0.049 (4)	0.036 (3)	0.094 (5)	−0.003 (3)	−0.009 (4)	−0.023 (3)
C27	0.043 (4)	0.051 (4)	0.107 (6)	−0.008 (3)	0.015 (4)	0.002 (4)
C28	0.041 (3)	0.036 (3)	0.066 (4)	0.002 (3)	0.007 (3)	0.000 (3)
N1	0.047 (3)	0.025 (2)	0.030 (2)	0.0021 (19)	0.0089 (19)	0.0011 (18)
N2	0.041 (3)	0.035 (2)	0.032 (2)	0.002 (2)	0.011 (2)	−0.0034 (19)
O1	0.107 (4)	0.040 (2)	0.066 (3)	0.005 (2)	0.040 (3)	0.018 (2)
O2	0.059 (3)	0.039 (2)	0.075 (3)	−0.012 (2)	0.003 (2)	−0.010 (2)
P1	0.0313 (7)	0.0320 (7)	0.0314 (7)	0.0006 (6)	0.0097 (5)	−0.0040 (6)

### Geometric parameters (Å, °)

**Table:**

C1—C6	1.385 (6)	C14—H14	0.9300
C1—C2	1.407 (6)	C15—C16	1.382 (9)
C1—P1	1.845 (5)	C15—H15	0.9300
C2—C3	1.374 (7)	C16—C17	1.360 (8)
C2—C7	1.505 (6)	C16—H16	0.9300
C3—C4	1.367 (7)	C17—H17	0.9300
C3—H3	0.9300	C18—N2	1.439 (6)
C4—C5	1.368 (7)	C18—H18A	0.9700
C4—H4	0.9300	C18—H18B	0.9700
C5—C6	1.366 (7)	C19—N2	1.463 (6)
C5—H5	0.9300	C19—C20	1.502 (8)
C6—H6	0.9300	C19—H19A	0.9700
C7—N1	1.456 (6)	C19—H19B	0.9700
C7—H7A	0.9700	C20—O2	1.420 (7)
C7—H7B	0.9700	C20—H20A	0.9700
C8—N1	1.444 (6)	C20—H20B	0.9700
C8—C9	1.491 (8)	C21—N2	1.454 (6)
C8—H8A	0.9700	C21—C22	1.496 (7)
C8—H8B	0.9700	C21—H21A	0.9700
C9—O1	1.418 (7)	C21—H21B	0.9700
C9—H9A	0.9700	C22—O2	1.423 (6)
C9—H9B	0.9700	C22—H22A	0.9700
C10—N1	1.465 (6)	C22—H22B	0.9700
C10—C11	1.499 (7)	C23—C24	1.385 (7)
C10—H10A	0.9700	C23—C28	1.385 (7)
C10—H10B	0.9700	C23—P1	1.841 (5)
C11—O1	1.413 (6)	C24—C25	1.379 (7)
C11—H11A	0.9700	C24—H24	0.9300
C11—H11B	0.9700	C25—C26	1.364 (8)
C12—C17	1.390 (7)	C25—H25	0.9300
C12—C13	1.407 (7)	C26—C27	1.384 (8)
C12—P1	1.842 (5)	C26—H26	0.9300
C13—C14	1.371 (7)	C27—C28	1.373 (8)
C13—C18	1.510 (7)	C27—H27	0.9300
C14—C15	1.383 (8)	C28—H28	0.9300
			
C6—C1—C2	118.1 (4)	C16—C17—C12	123.4 (6)
C6—C1—P1	123.6 (4)	C16—C17—H17	118.3
C2—C1—P1	118.2 (4)	C12—C17—H17	118.3
C3—C2—C1	118.7 (4)	N2—C18—C13	114.7 (4)
C3—C2—C7	120.9 (4)	N2—C18—H18A	108.6
C1—C2—C7	120.4 (4)	C13—C18—H18A	108.6
C4—C3—C2	121.9 (5)	N2—C18—H18B	108.6
C4—C3—H3	119.1	C13—C18—H18B	108.6
C2—C3—H3	119.1	H18A—C18—H18B	107.6
C3—C4—C5	119.8 (5)	N2—C19—C20	110.8 (5)
C3—C4—H4	120.1	N2—C19—H19A	109.5
C5—C4—H4	120.1	C20—C19—H19A	109.5
C6—C5—C4	119.5 (5)	N2—C19—H19B	109.5
C6—C5—H5	120.2	C20—C19—H19B	109.5
C4—C5—H5	120.2	H19A—C19—H19B	108.1
C5—C6—C1	122.0 (5)	O2—C20—C19	111.3 (5)
C5—C6—H6	119.0	O2—C20—H20A	109.4
C1—C6—H6	119.0	C19—C20—H20A	109.4
N1—C7—C2	113.0 (4)	O2—C20—H20B	109.4
N1—C7—H7A	109.0	C19—C20—H20B	109.4
C2—C7—H7A	109.0	H20A—C20—H20B	108.0
N1—C7—H7B	109.0	N2—C21—C22	110.7 (5)
C2—C7—H7B	109.0	N2—C21—H21A	109.5
H7A—C7—H7B	107.8	C22—C21—H21A	109.5
N1—C8—C9	110.2 (5)	N2—C21—H21B	109.5
N1—C8—H8A	109.6	C22—C21—H21B	109.5
C9—C8—H8A	109.6	H21A—C21—H21B	108.1
N1—C8—H8B	109.6	O2—C22—C21	110.9 (5)
C9—C8—H8B	109.6	O2—C22—H22A	109.5
H8A—C8—H8B	108.1	C21—C22—H22A	109.5
O1—C9—C8	112.5 (5)	O2—C22—H22B	109.5
O1—C9—H9A	109.1	C21—C22—H22B	109.5
C8—C9—H9A	109.1	H22A—C22—H22B	108.0
O1—C9—H9B	109.1	C24—C23—C28	118.2 (5)
C8—C9—H9B	109.1	C24—C23—P1	125.6 (4)
H9A—C9—H9B	107.8	C28—C23—P1	116.3 (4)
N1—C10—C11	109.9 (4)	C25—C24—C23	121.3 (5)
N1—C10—H10A	109.7	C25—C24—H24	119.4
C11—C10—H10A	109.7	C23—C24—H24	119.4
N1—C10—H10B	109.7	C26—C25—C24	119.7 (6)
C11—C10—H10B	109.7	C26—C25—H25	120.1
H10A—C10—H10B	108.2	C24—C25—H25	120.1
O1—C11—C10	112.1 (5)	C25—C26—C27	120.0 (6)
O1—C11—H11A	109.2	C25—C26—H26	120.0
C10—C11—H11A	109.2	C27—C26—H26	120.0
O1—C11—H11B	109.2	C28—C27—C26	120.1 (6)
C10—C11—H11B	109.2	C28—C27—H27	119.9
H11A—C11—H11B	107.9	C26—C27—H27	119.9
C17—C12—C13	116.4 (5)	C27—C28—C23	120.7 (6)
C17—C12—P1	124.4 (4)	C27—C28—H28	119.6
C13—C12—P1	119.1 (4)	C23—C28—H28	119.6
C14—C13—C12	119.9 (5)	C8—N1—C7	111.1 (4)
C14—C13—C18	119.0 (5)	C8—N1—C10	109.1 (4)
C12—C13—C18	121.1 (5)	C7—N1—C10	111.7 (4)
C13—C14—C15	122.5 (6)	C18—N2—C21	112.8 (4)
C13—C14—H14	118.8	C18—N2—C19	111.1 (4)
C15—C14—H14	118.8	C21—N2—C19	108.9 (4)
C16—C15—C14	117.9 (6)	C11—O1—C9	108.9 (4)
C16—C15—H15	121.0	C20—O2—C22	109.9 (4)
C14—C15—H15	121.0	C23—P1—C12	100.6 (2)
C17—C16—C15	119.9 (6)	C23—P1—C1	101.2 (2)
C17—C16—H16	120.0	C12—P1—C1	102.2 (2)
C15—C16—H16	120.0		
			
C6—C1—C2—C3	−0.1 (7)	C24—C25—C26—C27	0.3 (9)
P1—C1—C2—C3	−179.5 (4)	C25—C26—C27—C28	0.5 (10)
C6—C1—C2—C7	178.3 (4)	C26—C27—C28—C23	0.1 (9)
P1—C1—C2—C7	−1.2 (6)	C24—C23—C28—C27	−1.4 (8)
C1—C2—C3—C4	−0.8 (7)	P1—C23—C28—C27	179.5 (4)
C7—C2—C3—C4	−179.2 (5)	C9—C8—N1—C7	−180.0 (5)
C2—C3—C4—C5	0.8 (8)	C9—C8—N1—C10	−56.4 (6)
C3—C4—C5—C6	0.1 (8)	C2—C7—N1—C8	−169.7 (4)
C4—C5—C6—C1	−1.0 (8)	C2—C7—N1—C10	68.2 (5)
C2—C1—C6—C5	1.0 (7)	C11—C10—N1—C8	56.4 (6)
P1—C1—C6—C5	−179.6 (4)	C11—C10—N1—C7	179.7 (4)
C3—C2—C7—N1	−120.4 (5)	C13—C18—N2—C21	64.1 (6)
C1—C2—C7—N1	61.3 (6)	C13—C18—N2—C19	−173.4 (5)
N1—C8—C9—O1	58.5 (7)	C22—C21—N2—C18	−179.5 (4)
N1—C10—C11—O1	−58.3 (6)	C22—C21—N2—C19	56.6 (6)
C17—C12—C13—C14	−1.4 (7)	C20—C19—N2—C18	179.5 (5)
P1—C12—C13—C14	−177.5 (4)	C20—C19—N2—C21	−55.6 (6)
C17—C12—C13—C18	176.6 (5)	C10—C11—O1—C9	57.8 (7)
P1—C12—C13—C18	0.5 (6)	C8—C9—O1—C11	−57.9 (7)
C12—C13—C14—C15	2.1 (9)	C19—C20—O2—C22	−58.0 (6)
C18—C13—C14—C15	−176.0 (5)	C21—C22—O2—C20	58.9 (6)
C13—C14—C15—C16	−1.5 (10)	C24—C23—P1—C12	83.7 (5)
C14—C15—C16—C17	0.3 (10)	C28—C23—P1—C12	−97.4 (4)
C15—C16—C17—C12	0.3 (10)	C24—C23—P1—C1	−21.1 (5)
C13—C12—C17—C16	0.3 (8)	C28—C23—P1—C1	157.8 (4)
P1—C12—C17—C16	176.2 (5)	C17—C12—P1—C23	−16.0 (5)
C14—C13—C18—N2	−121.9 (5)	C13—C12—P1—C23	159.8 (4)
C12—C13—C18—N2	60.1 (6)	C17—C12—P1—C1	88.0 (5)
N2—C19—C20—O2	57.3 (7)	C13—C12—P1—C1	−96.2 (4)
N2—C21—C22—O2	−59.1 (6)	C6—C1—P1—C23	83.1 (4)
C28—C23—C24—C25	2.3 (8)	C2—C1—P1—C23	−97.4 (4)
P1—C23—C24—C25	−178.8 (4)	C6—C1—P1—C12	−20.4 (4)
C23—C24—C25—C26	−1.8 (9)	C2—C1—P1—C12	159.0 (4)

### Hydrogen-bond geometry (Å, °)

**Table:**

*D*—H···*A*	*D*—H	H···*A*	*D*···*A*	*D*—H···*A*
C25—H25···O1^i^	0.93	2.46	3.370 (8)	166
C11—H11B···O2^ii^	0.97	2.55	3.426 (6)	151

Symmetry codes: (i) *x*, −*y*+1/2, *z*−1/2; (ii) *x*, *y*−1, *z*.
